# Association Between Aspirin Use and Biliary Tract Cancer Survival

**DOI:** 10.1001/jamaoncol.2019.4328

**Published:** 2019-10-17

**Authors:** Sarah S. Jackson, Ruth M. Pfeiffer, Zhiwei Liu, Lesley A. Anderson, Huei-Ting Tsai, Shahinaz M. Gadalla, Jill Koshiol

**Affiliations:** 1 Division of Cancer Epidemiology and Genetics, National Cancer Institute, Rockville, Maryland; 2 Centre for Public Health, School of Medicine, Dentistry and Biomedical Science, Queen’s University Belfast, United Kingdom; 3 Office of Surveillance and Epidemiology, Center for Drug Evaluation and Research US Food and Drug Administration, Silver Spring, Maryland; 4 Lombardi Comprehensive Cancer Center, Georgetown University Medical Center, Georgetown University, Washington, DC

## Abstract

This database study analyzes the association between aspirin use and survival in patients with biliary tract cancer.

Biliary tract cancers (BTCs) are rare, with a worldwide incidence of less than 2 per 100 000 individuals.^1^ The 5-year survival rate ranges from 5% to 15%, with a median survival of less than 1 year.^1^ Between 60% and 70% of patients present with late-stage disease (eg, inoperable or metastatic tumors) owing to the lack of symptoms.^2^ Consequently, there is a critical need for treatments that improve BTC survival. Aspirin has been proposed as a treatment to reduce cancer mortality because it may slow cancer growth through the inhibition of both cyclooxygenase 2, which promotes inflammation and cell proliferation,^3^ and platelet aggregation, which may slow the metastatic spread of cancer.^4^ We investigated the association between postdiagnosis aspirin use and BTC survival.

## Methods

This study was approved by the National Institutes of Health Human Research Protection Program and the Independent Scientific Advisory Committee of the Clinical Practice Research Datalink ([CPRD] Protocol 17_160.R), and it was deemed exempt from patient written consent requirements because it was conducted using deidentified data. We obtained data, including all-cause deaths, on adult patients diagnosed with BTC from 1990 through 2017 from the United Kingdom’s CPRD, an electronic medical record database. We identified cancers using Read codes for gallbladder cancer (GBC), cholangiocarcinoma, ampulla of Vater cancer (AVC), and overlapping lesions of the biliary tract. We excluded patients with previous cancer, except for nonmelanoma skin cancer.

Ever use of postdiagnosis aspirin was defined as 1 prescription or more recorded in the CPRD on or after the BTC diagnosis date. We used Cox proportional hazards regression models to estimate the cancer site-specific hazard ratios (HRs) and 95% CIs for the association between time–dependent postdiagnosis aspirin use and overall survival. Patients who received an aspirin prescription within 30 days of diagnosis entered the model as users. The time scale began at diagnosis until death, exit from the study, or the end of follow-up (truncated at 10 years). We adjusted for the following covariates a priori: age at diagnosis, sex, comorbidities, statin use at diagnosis, indicators of a healthy lifestyle, and year of diagnosis. We fit separate models for each BTC type and stratified the baseline hazard by prediagnosis aspirin use (yes/no). We estimated adjusted survival curves using a marginal approach to remove the sex and age effects on aspirin use, accounting for the time-dependent exposure.^5^ We conducted analyses from April to May 2019 using SAS (version 9.4; SAS Institute) and survival curves in R Studio (version 1.1.453).

## Results

Among the eligible 2934 patients with BTC, 667 (23%) had GBC; 1559 (53%) cholangiocarcinoma; 224 (8%) AVC; and 484 (16%) overlapping. There were 2415 deaths (82%), with a median survival of 5.8 (interquartile range, 2-15) months. Two-hundred and fifty-six (9%) patients were aspirin users at baseline, with an additional 349 (12%) patients initiating aspirin use after diagnosis. Ninety-six percent of aspirin users (n = 2817) were prescribed a 75-mg dose. Compared with nonusers, aspirin users were more likely to be older, current statin users, and prediagnosis aspirin users and were more likely to have heart disease and comorbidities.

Aspirin use was associated with decreased risk of death in patients with GBC (HR, 0.63; 95% CI, 0.48-0.83), cholangiocarcinoma (HR, 0.71; 95% CI, 0.60-0.85), AVC (HR, 0.44; 95% CI, 0.26-0.76), and overlapping BTC (HR, 0.68; 95% CI, 0.50-0.92) (Table). The survival probabilities are shown in the Figure. Incident users with no history of aspirin use had a larger benefit from postdiagnosis aspirin use than prevalent users, although all users had a reduction in risk of death.

**Table.** Time-Dependent Associations Between Postdiagnosis Aspirin Use and Overall Survival for Each Biliary Tract Cancer Site^a^

	Gallbladder		Cholangiocarcinoma		Ampulla of Vater		Overlapping Lesions	
	No. of Events/No. at Risk^b^	HR (95% CI)	No. of Events/No. at Risk^b^	HR (95% CI)	No. of Events/No. at Risk^b^	HR (95% CI)	No. of Events/No. at Risk^b^	HR (95% CI)
**Overall**								
Nonusers	499/600	1 [Reference]	1198/1419	1 [Reference]	116/186	1 [Reference]	360/437	1 [Reference]
Users	54/67	0.63 (0.48-0.83)	123/140	0.71 (0.60-0.85)	26/38	0.44 (0.26-0.76)	39/47	0.68 (0.50-0.92)
**Prediagnosis Aspirin Use**								
Nonusers	145/145	1 [Reference]	383/383	1 [Reference]	33/33	1 [Reference]	119/119	1 [Reference]
Prevalent users^c^	49/50	0.69 (0.50-0.94)	114/114	0.78 (0.65-0.95)	26/26	0.41 (0.22-0.79)	37/38	0.76 (0.54-1.07)
**No Prediagnosis Aspirin Use**								
Nonusers	354/455	1 [Reference]	815/1036	1 [Reference]	83/153	1 [Reference]	241/318	1 [Reference]
Incident users^c^	5/17	0.57 (0.28-1.17)	9/26	0.37 (0.21-0.64)	0/12	0.21 (0.03-1.56)	2/9	0.34 (0.12-0.94)
*P* value for interaction^d^	.03		<.001		.005		.005	

Abbreviations: BTC, biliary tract cancer; HR, hazard ratio; NA, not applicable.

^a^ Adjusted for sex, history of heart disease, statin use (current, former, never), presence of comorbidities, age at diagnosis, and year of diagnosis. Aspirin use was modeled as time dependent and the baseline hazard was stratified by prediagnosis aspirin use.

^b^ The results presented used Cox regression where aspirin was modeled as time dependent (eg, individuals could switch between use and nonuse status). The numbers represent aspirin use at the time of BTC diagnosis.

^c^ Prevalent users were defined as patients with 2 or more aspirin prescriptions before BTC diagnosis. Incident users were defined as patients who only initiated aspirin use on or after the BTC diagnosis date.

^d^ *P* values for interaction were estimated by putting a cross-product term in the models for postdiagnosis use and prediagnosis use.

**Figure.**
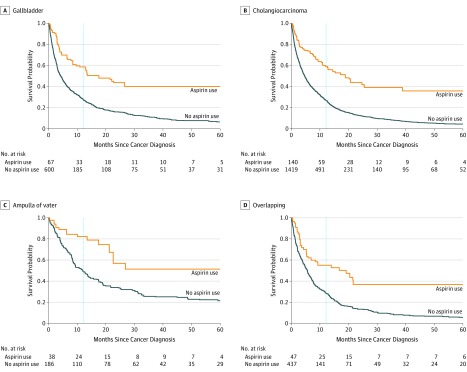
Adjusted Survival Curves Among Postdiagnosis Aspirin Users and Nonusers by Cancer Site Survival curves were weighted by age and sex distributions of the cohort with aspirin use modeled as time dependent. In patients with gallbladder cancer, the survival probabilities were for aspirin users vs nonusers were 59% (95 CI, 31%-100%) vs 27% (95% CI, 16%-47%). The survival probabilities in aspirin users vs nonusers with cholangiocarcinoma were 62% (95% CI, 42%-98%) 26% (95% CI, 19%-35%). In patients with ampulla of Vater cancer, the survival probabilities were 85% (95% CI, 33%-83%) vs 52% (95% CI, 18%-43%) in aspirin users vs nonusers, respectively. Survival probabilities in aspirin users vs nonusers with overlapping lesions of the biliary tract were 57% (95% CI, 34%-100%) vs 27% (95% CI, 16%-46%). Estimated survival at 1 year after diagnosis (dotted line) was calculated with 95% CIs computed based on the quantiles of the corresponding bootstrap distribution function with 1,000 replications. The numbers at risk represent individuals at the beginning of each time point.

## Discussion

We observed a reduced risk of death for postdiagnosis aspirin users across all BTC types. Platelet activation protects tumor cells from elimination, enhances metastatic cell growth, and enables cancerous cells to spread via the bloodstream.^4,6^ Aspirin may slow the metastatic spread of cancer cells through inhibition of platelet aggregation, improving BTC survival.^1^ A limitation of our analysis is the lack of data on cancer stage and chemotherapy regimens received (if any). However, most BTCs are diagnosed at late stage^2^ with less than 10% of patients presenting with resectable tumors and 50% of tumors metastasizing to the lymph nodes.^1^ The survival benefit of aspirin observed in our study is on par with the current standard of care.^2^
